# Dental Press Journal of Orthodontics and QUALIS**

**DOI:** 10.1590/2177-6709.20.5.012-013.edt

**Published:** 2015

**Authors:** David Normando

**Affiliations:** * Adjunct Professor, School of Dentistry, Universidade Federal do Pará (UFPA). Head of the post-graduation program in Dentistry, Universidade Federal do Pará and Specialization Course in Orthodontics, ABO-Pará.

Give me a lever long enough and a fulcrum on which to place it, and I shall move the world. Archimedes (Greek mathematician, physicist and astronomer)

A few years ago, the Dentistry section of CAPES (Brazilian Coordination for the Improvement of Higher Education Personnel) decided to induce the QUALIS^**^ of three scientific national dental journals, among which four were indexed at SciELO database. At that time, based on the criteria of the field, the three main Brazilian journals (BOR, JAOS and BDJ), which used to be classified as B2, were induced to be classified as B1. A decision to be loudly applauded. Why?

Whenever a given journal is ranked higher at QUALIS-CAPES, Brazilian researchers tend to give priority to it when submitting manuscripts for publication. A knock-on effect is then produced: more articles, better selection of manuscripts, higher impact of published articles and, as a result, higher impact factor for this journal. What was envisioned has been accomplished: in 2009, those three journals had a *Cites per document *index ranging from 0.49 to 0.69;^1^ after the induction mechanism, all three journals had their citation index doubled. Presently, the citation index of those three journals ranges between 0.91 and 1.04 at SCImago^1^. Brazilian science is often criticized for producing much and publishing little. Now, it found an excellent tool to boost the number of citations of its own periodicals. Why not?

In order to proceed with such development, in August, 2015, the Dentistry section of CAPES enhanced the arm of the lever and reinforced its fulcrum: all three journals were ranked as A2. Another major decision based on the development resulting from the first action and the need to polish our precious stones. 

At first, the fourth Brazilian dental journal indexed at SciELO that would have its QUALIS raised was Dental Press Journal of Orthodontics (DPJO). With an impact factor way below other national journals also indexed in the same database (at around 0.075) and due to being published in Portuguese only, we decided to make some progress before we were given the opportunity of undergoing the induction process, despite the enormous importance our journal has among Brazilian and foreigner orthodontists. 

As from 2010, DPJO began to be published in English and had its title changed. Since then, we had been doubling our impact factor which reached 0.25 in 2012. 

At that time, there was an avalanche of good articles. In 2013, however, growth had to slow down, as we decided to double the number of articles published with a view to speeding the process of publishing up. In 2014, DPJO *Cites per doc*partially regained the loss suffered in 2013 and reached an impact factor of 0.16. The future looks promising in 2015, with a well-adjusted publication flow and an estimate of the impact resulting from indexing at PubMed Central.

Despite the growth of the journal in the last few years, the Dentistry section of CAPES recently ranked DPJO as QUALIS B3. It is only natural that Brazilian Orthodontics questions the following: in spite of its growth and without the benefits provided by induction, what is the reason why DPJO was not ranked higher in the recent QUALIS evaluation conducted by CAPES?

A reasonable assumption is the fact that DPJO is a specialty journal, which is undeniable; however, a few particularities should be highlighted. Orthodontics is the only specialty of Brazilian Dentistry that counts on a journal indexed by the major databases worldwide. (SciELO, Scopus, PubMed/Medline and PubMed Central). According to SCImago, in 2014, Brazil was ranked second among the countries with the highest scientific production in Dentistry. The United States was ranked first, with 40% more scientific production than Brazil. The orthodontic reality is quite similar; however, the distance is shorter: 19%. Should we disregard Brazilian researchers' production published by DPJO, the scientific production of the country would decrease in 40% and Brazilian Orthodontics would be ranked eighth, with half as much production as the USA. Thus, DPJO has played a major role in disclosing Brazilian orthodontic science. 

Furthermore, despite being the lead journal in which Brazilian Orthodontics is disclosed, DPJO also publishes articles written by researchers focused on other dental as well as medical, engineering and speech therapy specialties, among others. In 2014, 86 out of 97 scientific articles published by DPJO were written by Brazilian researchers, and nearly as half (40) had professors from other specialties as coauthors. Therefore, data reveal that despite being a specialty journal, DPJO is highly receptive to other specialties but Orthodontics, which reinforces the multidisciplinary facet of the field.

An analysis of articles published between 2013 and 2014, the period on which the most recent CAPES evaluation was based on, reveals that students and professors attending 51 different graduate programs (53.6%) published at DPJO. This accounts for the majority of Brazilian graduate programs. The percentage is equivalent to the diversity reported by the journals benefiting from QUALIS induction process. Thus, inducing DPJO QUALIS would benefit the majority of Brazilian graduate programs, not only a few of them. It is paramount to highlight the substantial contribution given by DPJO to professors of Orthodontics as well as other specialties, allowing them to remain part of different graduate programs. Moreover, it is possible that its induction would also allow professors of Orthodontics to participate more actively in programs that do not have Orthodontics as one of their specialties, contributing to make Brazilian graduate programs diverse and enhancing multidisciplinarity in scientific production, which is paramount to increase the impact of Brazilian Dental Science.

David Normando - editor-in-chief (davidnormando@hotmail.com)

## References

[1] SCImago Journal Rank (SJR) (2009). Cites per document index - 2009.

